# Molecular engineering of lysosome-based degraders unveils a rapidly expanding therapeutic strategy

**DOI:** 10.1080/15548627.2026.2618626

**Published:** 2026-02-08

**Authors:** Adele Rivault, Jade Dussart-Gautheret, Rachid Benhida, Anthony R Martin, Patrick Auberger, Arnaud Jacquel, Guillaume Robert

**Affiliations:** a INSERM, Université Côte d’Azur, Nice, France; b CNRS, ICN UMR 7272, Université Côte d’Azur, Nice, France; c CNRS UMR 5247, ENSCM, Institut des Biomolécules Max Mousseron, Université de Montpellier, Montpellier, France

**Keywords:** Biodegraders, chimera compounds, drug design, Iysosome, targeted degradation

## Abstract

The targeted degradation of oncogenic or misfolded proteins has emerged as a promising therapeutic strategy. While proteolysis-targeting chimeras (PROTACs) and related technologies have successfully hijacked the ubiquitin-proteasome system to eliminate disease-driving proteins, recent advances highlight the lysosome as a powerful alternative degradation route. Lysosome-based degradation strategies offer broader substrate scope, subcellular targeting flexibility, and the ability to degrade proteins beyond the reach of the proteasome. In this review, we provide a comprehensive overview of synthetic molecules and engineered systems designed to traffic target proteins to the lysosome. These include lysosome targeting chimeras (LYTACs), autophagy-targeting chimeras (AUTACs), autophagy-tethering compounds (ATTECs), and other modalities that exploit endogenous trafficking pathways for selective protein clearance. By mapping the current landscape of lysosome-targeting degraders, this article underscores the therapeutic potential of lysosomal proteolysis and outlines future directions for molecular engineering in this rapidly evolving field.

## Introduction

Targeted protein degradation (TPD) has aroused growing interest and massive investment in recent years as a powerful approach in drug discovery, offering a solution to target proteins that have previously been considered “undruggable” [1]. Unlike traditional inhibitors that inhibit protein activity through occupancy-driven pharmacology, TPD aims to direct a protein of interest toward degradation using degradation effectors, thus achieving protein down-regulation and bypassing some limitations of conventional drug approaches. Initial development in this field focused on the ubiquitin-proteasome system (UPS), mainly with the advent of proteolysis-targeting chimeras (PROTACs) [2–7]. Since then, several PROTACs have entered clinical trials, highlighting their high potential in medicinal chemistry and chemical biology [8]. TPD’s potential has quickly expanded to include other cellular degradation pathways, opening-up new therapeutic possibilities. Among them, lysosomal degradation has shown great potential, especially for targeting proteins and aggregates that are difficult to address with proteasome. The TPD technology is now applicable to various diseases including cancer, metabolic disorders, neurodegenerative and genetic disease [9,10]. The lysosomal machinery is one of the cell’s two primary degradation routes and can process a wide range of targets, including both intracellular and extracellular proteins [11,12]. Unlike the proteasome, which mainly breaks intracellular, soluble, and ubiquitin-tagged proteins within the cytoplasm and nucleus [6], lysosomes degrade larger and more complex substrates, such as membrane-bound proteins, extracellular proteins, intracellular aggregates and even damaged organelles. Both pathways interact with each other to achieve compensatory or synergistic outcomes, but also contribute to ensuring the specificity of the degradation of target proteins in cells [13,14]. Such flexibility offers advantages in treating diseases in which protein buildup or misfolding plays a key role, including neurodegenerative diseases, genetic disorders and cancers [15]. The inherent capabilities of lysosomal degradation also offer new therapeutic possibilities for addressing protein targets in the extracellular environment or those associated with specific cellular compartments. Targeting proteins that are involved in pathological signaling at the cell surface or within distinct organelles could provide a new level of specificity providing solutions for diseases with limited treatment options. This opportunity has led to the development of lysosomal-based degraders engineered to exploit different entry points and pathways within the lysosomal system such as endosomal-based degraders including LYTACs, antibodies-based PROTAC (AbTACs), cytokine receptor-targeting chimeras (KineTAC), glue-targeted chimeras (GlueTAC)-like as well as degraders that target autophagy-related pathways e.g., AUTACs, AUTOTACs (autophagy-targeting chimeras), ATTECs (autophagy-targeting chimera) and chaperone-mediated autophagy (CMA)-based degraders. Indeed, the cardinal roles of lysosomes in protein and organelle degradation make them an appealing target for therapeutic intervention, as lysosomal degraders can potentially address a wide variety of disease-driver proteins that may otherwise not be accessible to a traditional drug discovery approach.

This review aims to provide a comprehensive overview of lysosomal-based degraders by discussing the chemical and structural diversity and functionality within lysosomal-endosomal-based degraders and autophagy-based degraders. First, we detail the different lysosomal-based degraded technologies that have emerged in recent years. We further describe the different ligand molecular classes and strategies used in the synthesis of these lysosomal-based degraders. Finally, we explore current advancements in the design strategies employed to enhance the specificity, stability, and activity of these molecules, including innovations such as molecular glues, trivalent compound, nanobodies, multitarget compounds, prodrugs and recent efforts to develop delivery technologies. We here highlight the challenges that remain in developing lysosomal-based degraders and the opportunities that lie ahead. It is essential to recognize the potential of lysosomal degradation to transform the landscape of targeted therapies in the future.

## Lysosomal degradation pathway

Lysosomes are ubiquitous acidic organelles and essential degradative centers in cells, discovered over 60 years ago by De Duve [16]. They contain multiple acid hydrolases and degrade diverse cellular components through multiple, pathway-specific mechanisms [17,18]. They are part of the proteostasis, responsible for maintaining the integrity, functionality, and balance of the proteome [19]. The lysosomal degradation is crucial for cell adaptation to different forms of stress through the management of damaged or misfolded proteins, thus regulating key processes such as signal transduction and immune response [17]. Disruptions in lysosomal function are associated with a plethora of diseases, including lysosomal storage diseases, neurodegenerative disorders, immune dysfunctions and cancers [11,12,20]. Lysosomal degradation is mediated through two primarily interconnected pathways: the endosomal-lysosomal pathway and autophagy. Both pathways share the lysosome as an indispensable effector, but differ significantly in their mechanisms of substrate delivery, targets, and cellular functions. Together, they form an extensive system for cellular clean-up and resource recycling [12,21].

### Endosomal-lysosomal degradation pathway

The endosomal-lysosomal pathway is cardinal to the degradation of extracellular proteins, membrane-associated molecules, and different pathogens. This pathway begins with induced proximity of a protein of interest with an effector protein that mediates endocytosis, where cellular material or extracellular components are internalized by invagination. Several steps of this pathway depend on the membrane-bound carrier or the coat protein of the vesicles, each driven by distinct mechanisms. Degradation via the endosomal-lysosomal system occurs through a coordinated sequence of membrane-bound intracellular compartments from early endosome, late endosome to the lysosome, through which the endocytosed materials flow [22].

The complexes formed between recruited extracellular component and targeted protein are generally internalized through clathrin-coated pits following the binding with transmembrane effectors proteins. Once taken up into cell, vesicles fuse with early endosomes, which serve as sorting hubs. In early endosomes, the cargo is next directed to one of the following destinations: it may be recycled to the plasma membrane or subjected to degradation. Proteins designated for degradation are sequestered into late endosomes and multivesicular bodies [23]. These structures eventually undergo a decrease in pH and mature into lysosomes [9]. The internalization of specific extracellular materials depends on the interaction between recognition motif-bearing cargo and their corresponding lysosome-shuttling receptor. This approach is referred to as receptor-mediated endocytosis, which is the main mechanism used by various degrader technologies [24,25]. Specific lysosomal receptors, such as IGF2R/CI-M6PR (insulin like growth factor 2 receptor) and ASGR (asialoglycoprotein receptor) recognize specific ligands such as mannose-6-phosphate (M6P), tri-N-acetylgalactosamine (GalNAc) or synthetic aptamers, ensuring precise delivery of cargo to lysosomes [26]. In addition, recent approaches proposed to mimic lysosomal sorting signals (LSS) to induce clathrin-mediated endocytosis without any lysosome-shuttling receptor requirement [27–29].

### Autophagy degradation pathway

Autophagy is a highly conserved cellular process that enables the degradation of various intracellular components including cytosolic proteins. It is critical for the maintenance of cellular proteostasis and the integration of stress responses triggered by nutrient deprivation [30] or oxidative damage [31,32]. The process is crucial for cellular protection as it eliminates damaged organelles and aggregated proteins, which could otherwise result in toxic effects [31]. In neurodegenerative disorders, for example, autophagy-based therapies are being explored to clear in particular accumulation of protein aggregates, which are not suitable for proteasome-dependent degradation due to their large size, such as mutant HTT (huntingtin) protein/mHTT or amyloid β, that contribute to disease pathology [33,34]. Unlike the endosomal-lysosomal pathway, which primarily handles extracellular and membrane-associated proteins, autophagy targets cytosolic material for degradation [35]. Autophagy proceeds through three main paths, each defined by the mechanism of delivery to lysosomes: Macroautophagy (MA), microautophagy (Mi) and CMA.

#### Macroautophagy (MA)

This is the most common and well-characterized form of autophagy. It begins with the generation of a double-membraned structure known as a phagophore, which elongates to encapsulate cytoplasmic material, forming an autophagosome. Once formed, the autophagosomes fuse with lysosomes, where their engulfed cytoplasmic contents are degraded. MA is highly selective, targeting organelles like mitochondria, long-lived proteins, protein aggregates, lipids [30], and even intracellular pathogens. MA is tightly controlled by a network of autophagy-related proteins that orchestrate the processes of autophagosome formation, elongation, and closure, in addition to facilitating their subsequent fusion with lysosomes [36]. The Atg8-family protein MAP1LC3/LC3 (microtubule associated protein 1 light chain 3), is a key player in autophagosome biogenesis and serves as an essential tether for engaging targets into the autophagy machinery. During phagophore expansion, LC3-I is converted to LC3-II and conjugated to a phosphatidylethanolamine (PE) by the ATG7 (autophagy related 7) and ATG3 proteins. This lipidation process enables LC3 to remain covalently attached to autophagosome membranes, making it a reliable marker of autophagosome biogenesis and a promising anchor for autophagy-based degrading technologies. Besides LC3, autophagy receptors like SQSTM1/p62 (sequestosome 1) are crucial in selective autophagy. These receptors identify ubiquitinated targets, link them to LC3, and facilitate their attachment to the phagophore, ultimately promoting fusion with lysosomes. Finally, K63-linked protein polyubiquitination is recognized by SQSTM1/p62 for selective autophagic degradation [37–40]. The regulated and selective characteristics of autophagy enable cells to respond effectively to varying metabolic requirements, shifts in environmental conditions, thereby maintaining cellular integrity and proteostasis [11,19].

#### Microautophagy (Mi)

In lysosomal microautophagy, lysosomes can directly engulf cytosolic components by the deformation of their membranes forming intraluminal compartments via internalization of lysosomal membrane proteins (LMPs) [41]. This process is less selective than MA but plays a significant role in general cytoplasmic turnover and quality control as it degrade cytoplasmic components and the membrane proteins within the invaginated portion of the membrane [42,43]. Lysosomal membrane proteins LMPs are short-lived and are degraded in the lysosome lumen in both a ubiquitin- and endosomal sorting complexes required for transport/ESCRT-dependent manner [44].

#### Chaperone-mediated autophagy (CMA)

CMA is a highly selective form of autophagy in which proteins containing a KFERQ-like motif are recognized by the cytosolic chaperone HSPA8/HSC70 (heat shock protein family A (Hsp70) member 8) and delivered to the membrane of lysosomes [45]. The KFERQ-like motif shows variability in its residue configuration, allowing substitutions with similar residues while maintaining recognition by HSPA8. Typically, the pentapeptide is flanked by a glutamine (Q) and includes one negatively charged residue (glutamic acid E or aspartic acid D), one/two positively charged residues (lysine K or arginine R), and one or two hydrophobic residues (phenylalanine F, valine V, leucine L, or isoleucine I). Additionally, post-translational modifications aimed at altering the charge of theses pentapeptide residues (e.g., phosphorylation or acetylation) may be beneficial, potentially enhancing motif recognition [9,46,47]. Upon reaching the lysosomal membrane, the complex consisting of the target protein and the chaperone HSPA8 interacts with lysosome-associated membrane protein type 2A (LAMP2A). Serving as the receptor for CMA, LAMP2A enables the translocation of these cytosolic proteins into the lysosome, by forming a homomultimeric complex that facilitates the translocation of the target protein that will subsequently be degraded in the intra-lysosomal lumen [36,48]. Approximately 40% of proteins in the mammalian proteome exhibit a KFERQ-like motif. This high prevalence of KFERQ-like motifs emphasizes the importance of CMA in cellular homeostasis and the regulation of various biological processes [49]. The lysosomal degradation pathway has vast therapeutic potential, with distinct roles for the endosomal-lysosomal and autophagy pathways. The former is particularly suited for targeting extracellular and membrane-bound proteins, making it ideal for diseases involving receptor dysregulation or extracellular protein buildup. The latter is well suited to address intracellular challenges, including organelle dysfunction and protein aggregation.

## Lysosomal-based degrader technologies

**Figure 1.**
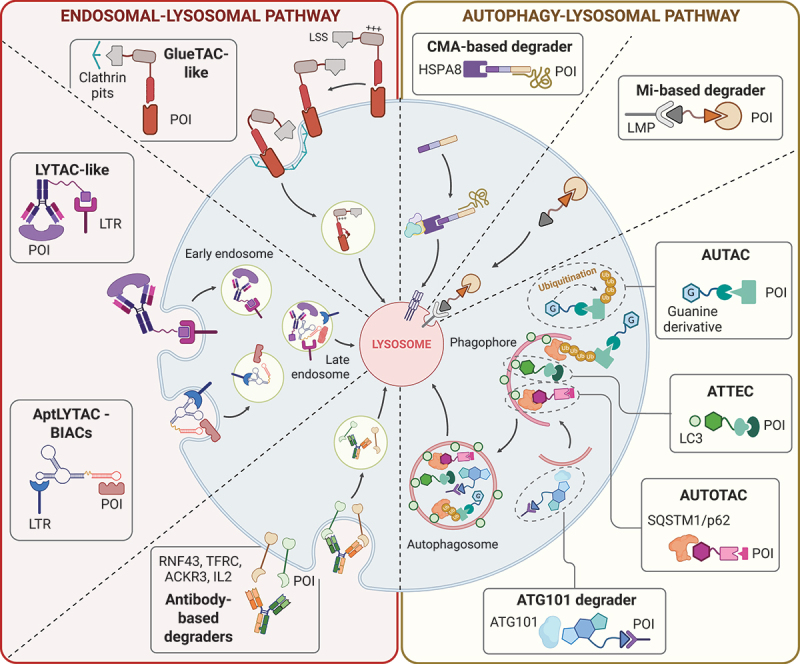
Representation of different lysosomal-based degraders and their pathway. On the one hand, LYTAC, GlueTAC-like, AptLYTAC, BIAC and antibody-based degrader promote POI degradation through endocytosis involving endosome formation. On the other hand, AUTAC, ATTEC, AUTOTAC and ATG101 recruiting degrader promote target degradation through MA involved in autophagosome formation; Mi-based degrader promotes lysosomal relocalization via microautophagy and CMA-based degrader promotes POI degradation through CMA.

Various types of lysosomal degrader with different mechanisms of action have been elaborated in recent years (Figure 1). TPD methodologies present significant benefits compared to traditional occupancy-based protein inhibition. A primary advantage is the extensive applicability of the target protein degradation approach, given its potential targets. Various lysosome-dependent TPD strategies have already been shown to effectively target membrane proteins, secreted proteins, and both intra- and extracellular protein aggregates [50]. It is essential to underline the potential to use low concentrations of degrader molecules, as the degradation process can be in some instances both catalytic and sub-stoichiometric [51]. This expanding range of degraders aimed to complement the PROTAC technology and offered a variety of targets, each having advantages and limitations (Table 1). PROTACs represent the most studied targeted-degradation modality, offering rapid degradation kinetics and a well-defined mechanism for eliminating soluble intracellular proteins [52]. Their utility, however, is shaped by intracellular accessibility, the dependence on specific E3 ligases, and their effectiveness mostly confined to soluble intracellular proteins. In contrast, lysosomal-based degraders that harness endocytic or autophagic pathways, enable the removal of substantially larger and more diverse complexes. These degraders are particularly advantageous in scenarios where the proteasome cannot efficiently process bulky, aggregated, or spatially inaccessible structures [53], though they depend on intact lysosomal machinery and in some cases still lack fully resolved mechanistic details [9]. Within this broad category, each lysosomal degrader technology offers distinct advantages and trade-offs. LYTACs, including antibody-based and aptamer-based versions, provide engagement of extracellular and membrane-bound proteins [50]. Autophagy-tethering systems such as AUTACs, ATTECs, and AUTOTACs expand the degradable substrate space to include aggregates, lipid droplets, damaged organelle, and other large intracellular proteins. However, they depend on basal autophagic flux and can be influenced by cell-state homeostasis. CMA-based degraders provide a selectivity for proteins bearing KFERQ-like motifs but are limited by the specificity and capacity of chaperone-mediated autophagy. Selecting an appropriate degradation strategy is dictated by target characteristics, as each technology whether PROTACs, LYTACs, or autophagy-based degraders are optimized for different target types and therapeutic contexts. While the development of lysosomal-based degraders has largely centered on cancer and neurodegenerative conditions, growing evidences highlight their potential in metabolic disorders (non-protein substrates lipid droplets [54]), hepatic contexts (liver-specific lysosome-targeting degrader [55,56]), and pathogens [57]. A summary of these different degrader technologies and their specific features, in particular their target lysosomal degradation receptor, target protein, and their different structure strategies, are presented in Tables 2 and 3.

**Table 1.** Different types of degraders, their target, advantages, limitations and therapeutic indication.

Degrader	Technology	Target	Degradation mechanism	Advantages	Limitations	Therapeutic indication & clinical maturity
Ubiquitin- proteasome pathway	PROTAC	Intracellular and membrane bound protein	Proteasome	Clear mechanisms, high selectivity, catalytic, fast kinetics	High hydrophobicity, E3-dependency	Phase III: ARV-471 (targeting ER for breast cancer), BMS-986365 (targeting AR for mCRPC), BGB-16673 (targeting BTK for B-cell malignancies)
Endosome-lysosome pathway	LYTACS-like	Extracellular protein; membrane protein	Lysosome targeting membrane-bound receptors (LTRs)	Tissue specificity depending on the LTR, high selectivity for targets	High molecular weight, poor tissue permeability, instability, LTR ligands synthetics sugars	Cancer, brain disorders, metabolic disorders Preclinical
	Aptamers-LYTAC	Extracellular protein; membrane protein		Aptamer high affinity	Low stability	Cancer, virus, ocular pathology Preclinical
	Antibody-based degrader	Membrane protein		Low immune response, can be recycled	High molecular weight, stability	Cancer Preclinical
	Clathrin-Mediated Endocytosis-Based Degrader	Membrane protein	Clathrin coat	High affinity and overcoming off-target effects	Low safety profile and uncertain half-life	Cancer Preclinical
Autophagy-lysosome pathway	AUTACS	Intracellular protein, damaged organelle	Ubiquitination via K63-TRAF6, SQSTM1/p62 binding	Wide degradation scope like organelles	Complex mechanism of action	Cancer, genetic disorders Preclinical
	ATTECS	Intracellular protein	LC3 binding	Broad target spectrum outside of proteins, chemical variety of ligand (even molecular glue)	Dependence on LC3 availability, potential off-target sequestration	Cancer, neurodegenerative disorders, metabolic disorders Preclinical
	AUTOTACS	Intracellular protein	SQSTM1/p62 binding	Wide degradation scope; no critical reliance on a specific length of linker	Only few investigations, classical chimera models	Cancer, neurodegenerative disorders Preclinical
	ATG101-recruiter degrader	Intracellular protein	ATG101 binding	Low molecular weight	Few investigation	Cancer Preclinical
	Mi-based degrader	Membrane-bound protein	LMPs binding	Target POI engagement via cytosolic side	Few investigations; less specific than other autophagy-based degradation	Cancer Preclinical
	CMA-based degrader	Cytosolic protein	HSPA8 binding	Rapid and direct degradation, wide degradation scope	Low safety profile, low stability	Cancer, ischemic stroke, neurodegenerative disorders Preclinical

**Table 2.** Recent endosomal-lysosomal-based degraders.

Year	Degrader technology	Binding protein for degradation	Type of degrader	Target	Target ligand type	Linker and strategy	Proof-of-concept stage & lead indication	Ref
2020	LYTAC	IGF2R	Chimera	APOE, EGFR, TFRC/CD71, CD274	Antibody	PEG4-N3 and BCN click chemistry coupling	*In cellulo* Cancer	[58]
2021	LYTAC (GalNAC-LYTAC)	ASGR	Chimera	EGFR, ERBB2	Antibody or c-integrin-binding peptide (PIP)	N3 and DBCO click chemistry coupling	*In cellulo* Cancer	[55]
2021	LYTAC (MoDE-As)	ASGR	Chimera	α-DNP, MIF	Small molecule	Linker PEG click chemistry coupling	*In vivo* Inflammatory diseases in hepatocyte	[56]
2021	LYTAC	ASGR	Chimera	NeutrAvidin	Small molecule	PEG linker	*In cellulo* Proof-of-concept in hepatocyte	[59]
2024	LYTAC (GLTAC)	GPC3	Chimera	CD274, c-MET, FGFR1	Small molecule/Peptide	GPC3 – targeting peptide – short alkyl linker, Click chemistry coupling	*In cellulo* Hepatocellular carcinoma, adenocarcinoma	[62]
2025	LYTAC (PepTAC)	TFRC	Chimeric peptide	CD274	Peptide+ Warhead	Covalent ligand-binding with aryl sulfonyl fluoride groups-CD274 antibody	*In vivo* Cancer, brain disorders	[61]
2025	LYTAC (SupraLYTAC)	CA9	Self-assembling peptide	extracellular protein and CD274	Peptide	Two amphiphilic peptides self-assembling into nanofibers	*In vivo* Cancer	[63]
2025	LYTAC (NanoCLY)	IGF2R	Peptide	CTGF	Peptide	Aliphatic chain-disulfide cleavable linker – CTGF targeting motif – M6P peptide; self-assembling nanoplatform	*In vivo* Triple-negative breast cancer	[60]
2025	HerTAC	ERBB2	Chimera	CD274, MIF, VSIR/VISTA	Small molecule	ERBB2-stapled peptide, Click chemistry, alkyl linker	*In vivo* Cancer	[64]
2021	Aptamer LYTAC	IGF2R	Chimera	MET and PTK7	Aptamer	DNA sequences synthesis all in one step	*In cellulo* Cancer	[67]
2023	Aptamer LYTAC (Apt-LYTAC)	ASGR	Chimera	PDGF and PTK7	Aptamer	Phosphoramidite method through solid phase synthesis	*In cellulo* Cancer	[68]
2023	Aptamer LYTAC	IGF2R	Chimera	CD274	Aptamer	Click chemistry coupling, optimization of linker length (6T base), covalent binding to POI	*In vivo* Cancer	[70]
2023	Aptamer LYTAC (HER2-LYTAC)	membrane-bound E3 ligase IGF2R	Chimera	ERBB2	Aptamer	Double-stranded DNA linker	*In cellulo* Breast cancer	[72]
2024	Aptamer LYTAC (multivalent AptLYTAC)	IGF2R	Trivalent compound	Simultaneously PTK7 and MET	Aptamer	Platform modulating aptamer/target ligand ratio using streptavidin and biotin synergy strategy	*In cellulo* Cancer, virus	[57]
2024	Aptamer LYTAC (VED-LYTAC)	IGF2R	Chimera	VEGF	Aptamer	Double-stranded DNA linker	*In vivo* Neovascular ocular disease	[71]
2024	Aptamer LYTAC (LYTAC Plus)	IGF2R	Trivalent design encapsulated in hydrogel	simultaneously VEGFR2 and ANGPT2/ANG2	Aptamer	Self-assembled Y-motif: M6P aptamer – dsDNA linker – VEGFR binding peptide – anti-*ANGPT2* siRNA, click chemistry coupling	*In vivo* Neovascular macular degeneration	[69]
2024	Aptamer LYTAC (Logic-TAC)	IGF2R	Chimera within an inactive form	MUC1	Aptamer	Membrane-guided DNA anchoring to a cancer cell indicator protein	*In vivo* Breast cancer	[73]
2021	AbTAC	Cell surface E3 ligase RNF43	Recombinant bispecific antibody	CD274	Antibody	Assembly of separately expressed half IgGs	*In cellulo* Breast cancer	[75]
2023	KineTAC	ACKR3 or IL2	Bispecific antibody	VEGF, TNF, CD274, ERBB2, EGFR, PDCD1, CDCP1, TACSTD2/TROP2	Nanobody	One arm for POI, the other for lysosome-targeting receptor	*In cellulo* Cancer	[76]
2024	TransTAC	TFRC	Bispecific antibody	EGFR, CD274, MS4A1/CD20, chimeric antigen receptor	Antibody	Two anti-TFRC – GFLG cathepsin-sensitive linker – two anti-POI	*In vivo* Lung cancer	[77]
2021	GlueTAC	AP-2/clathrin-coat	Nanobody	CD274	Nanobody	CPP – LSS – proximity reactive uncanonical amino acid to covalently binds target protein	*In vivo* Cancer	[28]
2023	SignalTAC	clathrin-coat	Tetravalent nanobody	ERBB2, EGFR CD274, MS4A1 and TFRC	Nanobody	CPP – LSS	*In vivo* Cancer	[27]
2024	PSMLTAC	AP-2/clathrin-coat	Chimera	CD274, PDE6D/PDEδ, NAMPT, BTK	Small molecule	CPP – LSS peptide coupled to a small molecule	*In cellulo* Cancer	[29]

DBCO: dibenzocyclooctyne; CPP: cell-penetrating peptide; LSS: lysosome sorting sequence.

**Table 3.** Recent autophagy-lysosomal-based degraders.

Year	Degrader technology	Binding protein for degradation	Type of degrader	Target	Target recognition type	Linker and strategy	Proof-of-concept stage and Lead indication	Ref
2019 –2020	AUTAC	K63, SQSTM1/p62	Chimera	METAP2, FKBP1A/FKBP12, BRD4, mitochondria and TSPO	Small molecule	PEG linker, peptidic coupling	*In cellulo* Cancer, genetic disorders	[81]
2024	AUTAC (Biguanide-AUTAC)	K63, SQSTM1/p62	Chimera	Mitochondria	Small molecule	Alkyl linker, peptidic coupling	*In cellulo* Cancer	[83]
2024	AUTAC (Nano-AUTAC)	K63, SQSTM1/p62	Nanoparticle of chimera + methotrexate	IDO	Small molecule	Supramolecular obtained by self-assembling pairing chimera and methotrexate via hydrogen bond	*In vivo* Cancer	[84]
2025	AUTAC (GPX4-AUTAC)	TRAF6, SQSTM1/p62	Chimera	GPX4	Small molecule	PEG linker, Click coupling reaction	*In vivo* Breast cancer	[86]
2025	AUTAC (Tau-AUTAC)	K63, SQSTM1/p62	Chimera	MAPT	Small molecule	short linker length	*In vivo* Alzheimer disease	[85]
2019–2020	ATTEC	LC3	Molecular glue	mHTT	Small molecule		*In vivo* Huntington disease	[33,87]
2021	ATTEC-like	LC3	Chimera	BRD4	Small molecule	PEG linker	*In cellulo* Cancer	[89]
2023	ATTEC	LC3	Chimeric peptide	USP30	Peptide	Peptide based ATTEC approach PBD-LC3-interacting region (LIR)-CPP	*In cellulo* Parkinson disease	[98]
2021	ATTEC (LD-ATTEC)	LC3	Chimera	LDs	Small molecule	alkyl linker; SN	*In vivo* Hepatic lipidosis	[54]
2022	ATTEC (NAMPT ATTEC)	LC3	Chimera	NAMPT	Small molecule	Alkyl linker SN + peptide coupling	*In cellulo* Cancer	[90]
2023	ATTEC (mT1)	LC3	Chimera	Mitochondria	Small molecule	Alkyl linker	*In cellulo* Neurodegenerative diseases	[91]
2023	ATTEC (ATACC)	LC3	Chimeric peptide	SNCA	Peptide	PBD- Linker- LC3 interacting region (LIR)-CPP	*In vivo* Neurodegenerative diseases	[99]
2023	ATTEC (ATNC)	LC3B	Chimera	HE4	Nanobody	Nanobody fused to LC3B protein: HE4 nanobody-LC3B-Cys-cyclic cell-penetrating peptide cR10*	*In cellulo* Ovarian cancer	[100]
2023	ATTEC	LC3	Chimera	SNCA	Aptamer	click chemistry coupling, alkyl linker	*In cellulo* Parkinson disease	[93]
2024	ATTEC	LC3	Chimera	PDE6D/PDEδ	Small molecule	alkyl linker, peptidic coupling	*In cellulo* Cancer	[92]
2025	ATTEC (SHP2 ATTEC)	LC3	Chimera	PTPN11/SHP2	Small molecule	Rational drug design strategy, PEG linker	*In vivo* Pancreatic ductal adenocarcinoma	[94]
2025	ATTEC (Nano-ATTEC)	LC3	Polymeric micelle of diblock polymers	BRD4, AR	Small molecule	Polymeric micelle self-assembly of 3 polymers, POI-ligand- PEG-b-PCL, LC3-ligand- PEG-b-PCL and pH-responsive PAE-b-PCL	*In cellulo* Cancer	[95]
2025	ATTEC	LC3	Chimera	Protein complexes CDK9-CCNT1, PRC2, CDK2- CCNA2, CCNE1, CDK4, CDK6-CCND1	Small molecule	SAR on LC3 recruiter Short (2 carbons) linker	*In cellulo* Cancer	[96]
2025	ATTEC (R406)	LC3	Molecular glue	SNCA	Small molecule	high-throughput screening via small-molecule microarray	*Ex vivo* Parkinson disease	[88]
2025	ATTEC	LC3	Chimera	Intracellular and Extracellular PCSK9	Small molecule	PEG ligand SN last step	*In vivo* Atherosclerosis	[97]
2022	AUTOTAC	ZZ domain of SQSTM1/p62	Chimera	ESR2, METAP2, AR, misfolded protein aggregates MAPT^P301L^	Small molecule	PEG linker, peptidic coupling	*In vivo* Cancer, neurodegenerative diseases	[101]
2023	AUTOTAC (ATC)	ZZ domain of SQSTM1/p62	Chimera	SNCA aggregates	Small molecule	PEG linker	*In vivo* Parkinson disease	[102]
2025	AUTOTAC	SQSTM1/p62	Chimera	AR and AR-v7	Small molecule	PEG linker	*In vivo* Prostate cancer	[104]
2025	ATG101-recruiting degrader	ATG101	Chimera	CDK9, CCNT1	Small molecule	S-indacene hydrophobic tag; short alkyl linker	*In vivo* Cancer	[106]
2025	Mi-based degrader (LYMTAC)	LMPs (RNF152, LAPTM4a, LAPTM5)	Chimera	Membrane protein KRAS	Small molecule	PEG linker, synthesis last step: peptidic coupling or click chemistry	*In cellulo* Cancer	[107]
2014	CMA degrader	HSPA8	Chimeric peptide	Native DAPK1, SNCA, DLG4/PSD-95	Peptide	CMPD (TAT)-PBD-CTM (sequence KFERQKILDQRFFE)	*In vivo* Ischemia, Parkinson’s disease	[108]
2019	CMA degrader (PD-LYSO)	HIP1R	Chimeric peptide	CD274/PD-L1	Peptide	PBD-epitope tag-HIP1R sorting signal (sequence MDFSGLSLIKLKKQ)	*In cellulo* Cancer	[113]
2019	CMA degrader	HSPA8	Chimeric peptide	CDK5	Peptide	CMPD(TAT)-PBD-CTM (sequence KFERQKILDQRFFE)	*In vivo* Ischemic stroke	[114]
2020	CMA degrader	HSPA8	Chimeric peptide	Amyloid β oligomers	Peptide	CMPD(TAT)- PBD- CTM (sequence KFERQKILDQRFFE)	*Ex vivo* Alzheimer disease	[34]
2020	CMA degrader (CMATAC)	HSPA8	Chimeric peptide	ESR1/Erα	Peptide	CMPD(TAT)- PBD- CTM (sequence KFERQKILDQRFFE)	*In cellulo* Breast cancer	[115]
2024	CMA degrader (SM-CMAD)	HSPA8	nanoparticle with a core peptidic unit	ESR1, AR, MAP2K1/MEK1, MAP2K2/MEK2, and BCR-ABL	Small molecule	Self-assembled peptide-based nanoparticle diphenylglycine-R R – CTM (KFERQ)	*In cellulo* Cancer	[111]
2024	CMA degrader (Ab-CMA)	HSPA8	Chimera	EGFR, CD274, and ERBB2	Antibody	CTM (sequence KFERQKILDQRFFE) added to monoclonal antibodies via Click chemistry cycloaddition	*In vivo* Cancer	[110]
2024	CMA degrader (InCMATAC)	HSPA8	Chimeric peptide	STAT3	Peptide	Peptide encapsulated in lipid nanoparticle: PBD-linker (E)-CTM (sequence KFERQKILDQRFFE)	*In vivo* Cancer	[112]

PEG: polyethylene glycol; PBD: protein-binding domain; SN: nucleophilic substitution; LDs: Liquid droplets; cR10*: cyclic cell-penetrating deca-arginine; PEGbPCL: poly(ethylene glycol)blockpoly(εcaprolactone); SAR: structure-activity relationship; CMPD: cell membrane-penetrating domain; CTM: CMA-targeting motif.

### Endocytosis-based degraders

#### LYTACs-like: LTR-based degrader technologies

Lysosome-targeting chimeras, or LYTACs, represent the first class of degraders that use receptor-mediated endocytosis to direct extracellular or membrane proteins to the lysosome and consequently mediate endosomal-lysosomal degradation. LYTACs are built on a bifunctional degrader blueprint and consist of a target-binding moiety (the nature of which may vary, peptide, antibody or small molecule) linked to a glycan (polypeptide) ligand that can bind to the cell-surface lysosome-shuttling receptor. Engineered by The Bertozzi group, LYTACs, by binding to cell surface lysosome targeting receptors (LTRs) such as IGF2R (M6P-LYTAC) [58] or ASGR (GalNAc-LYTAC) [55], facilitate the internalization and lysosomal degradation of their targets. This approach, which has since been adopted by other laboratories [55,56,59,60], is particularly effective for targeting membrane-bound and extracellular proteins such as MIF (macrophage migration inhibitory factor), CD274/PD-L1 (CD274 molecule), and EGFR (epidermal growth factor receptor). New LTRs have been proposed recently such as TFRC (transferrin receptor), GPC3 (glypican 3), CA9/CAIX (carbonic anhydrase 9) and ERBB2/HER2 (erb-b2 receptor tyrosine kinase 2) [61–64]. These new LTRs offers a unique therapeutic avenue for cell surface proteins implicated in cancer, immune diseases, and other disorders. Ultimately, the selection of a specific lysosome targeting receptors, such as ASGR and GPC3 can offer an organ-specific targeting and have a significant impact on cell-selectivity and efficacy of lysosomal degraders [55,56,59,62]. The LTR can also display high recycling efficiency, like the TRFC receptor that actively degrade the target protein [61,65]. Finally, a spatiotemporally controlled LYTAC has been reported using a photothermal inducible switch expanding the way to control drug activity and release [66].

#### AptLytacs and BIACs

While antibody-based LYTACs can degrade a broad range of proteins of interest, the conjugation process between the antibody and polypeptide molecules is complex and associated with high manufacturing costs. High-molecular-weight LYTAC chimeras often exhibit low internalization efficiency. To address this, aptamer-based LYTAC (AptLYTAC) [67] was developed, which uses aptamers as the binding moiety for the target protein. Aptamers are short DNA or RNA sequences with high binding affinity, providing advantages like smaller size, increased stability, and simpler synthesis compared with antibodies. AptLYTACs are composed of an aptamer as the protein of interest (POI) binding element and a glycosylated polypeptide (M6Pn polypeptide or GalNAC) to recruit endosome shuttling receptors LTRs [57,68]. This dual-targeting mechanism allows AptLYTACs to internalize endogenous extracellular and membrane-bound proteins such as transmembrane receptors and guide them to lysosomes for degradation. Additionally, BIACs (bispecific aptamer chimeras) have been designed, where both the POI ligand and lysosome shuttling receptors ligand (for both IGF2R and ASGR) are aptamers. These aptamers used double-stranded DNA as a spacer to bridge and help with the stabilization of the whole degrader construct, resulting in lysosomal degradation of complex of interest. The AptLYTAC-BIAC concept has attracted significant attention in recent years [69–73] due to its ability to bind with high affinity to key proteins, including PDGF (platelet derived growth factor), VEGF (vascular endothelial growth factor), and CD274/PD-L1. This high-affinity binding lends them appeal for a wide variety of applications [74]. Variants like antibodies-based PROTAC (AbTACs) and glue-targeted chimeras (GlueTACs) extend the range of LYTAC applications, incorporating new features such as covalent binding or antibody specificity.

#### Antibody-based degraders: AbTACs, KineTACs and TransTAC

The AbTAC approach involves the use of recombinant bispecific antibodies to recruit RNF43 (ring finger protein 43), a single-pass transmembrane E3 ligase with one arm and cell-surface target proteins with the second, prompting their internalization and ultimately their lysosomal degradation [75]. While conceptually related to PROTACs, AbTACs leverage a mechanism like lysosome-targeting chimeras (LYTACs) by recruiting RNF43 to internalize cell-surface protein. This enables endosomal-lysosomal degradation rather than proteasomal pathways. Like AbTACs, cytokine receptor-targeting chimeras (KineTACs) and TFRC targeting chimeras (TransTACs) are bispecific antibodies able to address membrane-bound proteins to the lysosome. They are distinct from AbTACs as they are resorting to alternative LTR like ACKR3 (atypical chemokine receptor 3) [76], IL2 (interleukin 2) cytokine receptors [76], or TFRC [77]. Moreover, they were also reported for their ability to degrade extracellular proteins in addition to the membrane-bounds. KineTACs relies on bispecific antibodies, engineered using recombinant antibody techniques such as the “knobs-into-holes” method [78], to combine two distinct functional domains. One domain binds to the target protein, while the other interacts with receptors responsible for internalization, ensuring efficient lysosomal delivery and degradation. Recently, TransTAC technology introduced a heterobifunctional antibody that facilitates the close proximity of TFRC and the target protein on the cell surface and within the early endosomes, a cathepsin-sensitive linker that can be cleaved, thereby releasing the POI from TFRC to proceed into the lysosomal degradation pathway [77]. This receptor polyvalency proposed by this different technologies AbTAC, KineTAC and TransTAC extend the scope of protein degradation to include transmembrane proteins, making them highly polyvalent for a wider range of therapeutic applications.

#### GlueTAC-like: clathrin-mediated endocytosis-based degrader technologies

A recently introduced class of lysosomal degraders exploit the clathrin-mediated endocytosis pathway to deliver target proteins to the lysosome for degradation. Central to this approach is the lysosome sorting sequence (LSS), a main peptidic sequence that interacts with elements of clathrin coats, facilitating the internalization and relocation of the complex of interest to the lysosome [79]. GlueTAC technology firstly developed this approach, employing a covalent nanobody-based design that incorporates the LSS for efficient lysosomal targeting [28]. Each GlueTAC molecule consists of three primary components: a covalent nanobody that binds specifically to cell-surface complex of interest, a cell-penetrating peptide (CPP) to enhance cellular entry, and the LSS, which directs the complex to the lysosome. This combination ensures precise targeting and degradation of membrane proteins while minimizing off-target effects. GlueTACs offer significant advantages for therapeutic applications, particularly for membrane proteins, due to their high binding specificity and efficient lysosomal routing. Building on this concept, signal-mediated lysosome-targeting chimera (SignalTAC) technology has introduced a versatile approach to membrane protein degradation [27,80]. By attaching functional LSS-CPP peptide motif to a target-specific binder, SignalTAC creates functional degrader ranging from small peptides to larger complex with antibody with a molecular mass higher than 100 kDa. This simplicity makes SignalTACs adaptable to various target types and molecular sizes. A following degrader was developed as a peptide-mediated small molecule chimeras (PSMLTACs) [29], which integrate peptide scaffolds to degrade both membrane-bound and intracellular proteins. PSMLTACs represent a highly effective and wide-ranging approach for the targeted degradation of proteins, demonstrating greater efficiency compared to the corresponding PROTAC compounds [29].

### Autophagy-based degraders

#### AUTACs

Autophagy-targeting chimeras (AUTACs), are the first TPD technology that harnesses the cell’s autophagy machinery to selectively degrade cytosolic proteins, nuclear localized proteins, and even fragmented organelles. AUTACs achieve this by tagging proteins with a guanine derivative, a tag mimicking S-guanylation recognized that induces K63-linked polyubiquitination. This signal is detected by the autophagy receptor SQSTM1/p62, which then transports the protein to autophagosomes for subsequent degradation via LC3 linkage. Each AUTAC molecule comprises a ligand for the protein of interest and a guanine derivative degradation tag – often a fluorobenzylguanine/FBnG group – that stimulates K63 polyubiquitination. Originally developed by the Arimoto group in 2019 [81], AUTACs have shown effectiveness in degrading cytosolic proteins and damaged mitochondria. These autophagy-based degraders, a concept adopted by various laboratories [82–85], present a novel way to target disease-linked intracellular components beyond the capabilities of proteasomal or LYTAC-based degraders using a tag mimicking S-guanylation to trigger triggering K63-linked polyubiquitination. Finally, Gong et al. proposed to target another polyubiquitination promoter protein, TRAF6 (TNF receptor associated factor 6), expanding the range of AUTACs which will allow its subsequent recognition by SQSTM1/p62 and degradation via autophagosome sequestration [86]. Nevertheless, the multi-step autophagic pathway may introduce complexity to their design and application.

#### ATTECs

Another promising technology within the field of autophagy-lysosomal-based degraders, proposed by the Lu group involves autophagosome-tethering compounds (ATTECS). ATTECS are small molecules that harness autophagy pathway by selectively tethering the POI to the phagophore [33,87]. ATTECs are molecular glue [33,88] or heterobivalent ligands that specifically bind to both MAP1LC3/LC3 (microtubule associated protein 1 light chain 3), a protein associated with autophagosome formation, and the POI, facilitating the autophagic degradation of the latter. This strategy has been particularly effective for reducing levels of disease-causing protein aggregates, nuclear located proteins, and mitochondria [88–97]. ATTEC technology has also been extended to degrade non-protein targets, such as lipid droplets (LDs) [54], which are implicated in metabolic disorders. Further developments have led to the elaboration of non-small molecule-based ATTEC compounds. These are exploiting either a peptide-based strategy [98,99], nanobody-based strategy [100] or mixed polymers consisting of a synthetic ligand linked to an hydrophilic PEG-b-PCL [95]. Overall, this LC3-targeting approach highlights ATTECs’ potential to target a broad range of molecules through autophagy, positioning them as a valuable tool for treating complex diseases.

#### AUTOTACs

Autophagy-targeting chimeras (AUTOTACs), developed as an autophagy-targeting chimera technology, also exploit the cell’s autophagy machinery but do so by binding to SQSTM1/p62, a major autophagy receptor [101–104]. Each AUTOTAC molecule contains two parts: an autophagy-targeting ligand that binds to the ZZ domain of autophagy cargo receptor SQSTM1/p62 and a target-binding ligand specific to the protein or complex to be degraded. Binding to SQSTM1/p62 initiates autophagosome formation by recognition of LC3, leading to the encapsulation and lysosomal degradation of the target. AUTOTACs have demonstrated versatility in degrading intracellular soluble proteins and misfolded aggregated proteins with high efficacy. This approach has shown promise in neurodegenerative diseases such as with MAPT/Tau degradation in Alzheimer disease [105] and cancer models [104], underscoring the broad utility of AUTOTACs within the autophagy-lysosomal pathway. Notably, AUTOTACs are less sensitive to linker length than other TPD strategies, allowing for more flexible and straightforward design [101].

#### ATG101-recruiting degrader

ATG101-recruiting degraders represent a novel class of autophagy-inducing molecules that leverage the cell’s autophagy degradation machinery such as AZ-9 [106], which functions by engaging ATG101, a component of the ULK1 (unc-51 like autophagy activating kinase 1) complex responsible for initiating autophagosome formation. This recruitment triggers the early steps of the autophagy-lysosome pathway, leading to the formation of autophagosomes through the involvement of LC3. Once the phagophore engulfs its cargo, it fuses with lysosomes, resulting in the degradation of target proteins such as CDK9 (cyclin dependent kinase 9) and CCNT1 (cyclin T1).

#### Mi-based degrader

Microautophagy-based degraders, featured in the recently introduced lysosome-membrane-targeting chimeras (LYMTACs) [107], are heterobifunctional small molecules that hijack shortlived lysosomal membrane proteins (LMPs) as effectors to carry across target proteins to the lysosome for degradation. Operating intracellularly, these degraders can redirect a range of membrane proteins, both integral and membrane-anchored, to lysosome by coupling them to LMPs such as RNF152 (ring finger protein 152), LAPTM4A (lysosomal protein transmembrane 4 alpha), or LAPTM5 (lysosomal protein transmembrane 5) [44], thereby inducing relocalization and subsequent lysosomal degradation. Unlike antibody-based LYTACs, LYMTACs leverage a chimera that engages targets from the cytosolic side, expanding the scope of degradable membrane proteins while retaining the specificity of proximity-inducing modalities.

#### CMA-based degraders

Chaperone-mediated autophagy (CMA) offers a selective pathway for lysosomal degradation, targeting soluble cytosolic proteins bearing a KFERQ-like motif [108] originally developed from empirical results [45]. Chaperone-mediated autophagy targeting chimeras (CMATACs) capitalize on this selectivity by incorporating KFERQ-mimetic peptide sequence into a bifunctional peptidic scaffold, enabling specific proteins to be delivered directly to the lysosome without the need for autophagosome formation using a structure divided into 3 functional domains: a CMA-targeting motif (CTM), a cell membrane penetrating domain (CMPD) and a protein binding domain (PBD). The most recurrent CMA-targeting motif (CTM) used in the development of several compounds is the KFERQKILDQRFFE sequence [109]. These degraders utilize the chaperone protein HSPA8, which guides tagged proteins to LAMP2A on the lysosome membrane for translocation into the lysosome lumen and degradation. Although still emerging, CMA-based degraders hold significant potential for targeting intracellular proteins and amyloid oligomers proteins implicated in diseases such as neurodegeneration and cancers [34,110–115]. The simplicity and straightforwardness of CMA make it an attractive mechanism. More recently, efforts have been made to improve and allow the passage through the membrane [111,112], and so as to diversify the structures of the degraders by using an antibody as a ligand for the target protein [110].

## Chemical diversity

Despite the promising potential of lysosomal-based degraders, their development is complex and requires a deep understanding of both chemical and biological principles. Effective lysosomal targeting relies on carefully designed chemical structures that can both bind to the protein of interest and engage with lysosomal pathways, which are often more selective and context-dependent than other cellular degradation mechanisms like proteasome-dependent degradation. Achieving such targeted degradation requires precision in the design of the binding domains and linker chemistry, as well as a comprehensive understanding of lysosomal receptors and entry points within the cell. The aptitude of TPD to facilitate degradation is significantly influenced by the interaction between the complex of interest and its corresponding ligand. The molecular class of various lysosomal-based degraders has been listed above in Tables 2 and 3 with an emphasis on novel designs and chemical diversity. Thereby, it can be determined that four main chemical entities are used as target recognition motifs: small molecules, peptides, antibodies, and aptamers (Figure 3).

Some designs are better suited for certain degradation pathways i.e., antibodies can be utilized for endosomal-lysosomal-based degraders such as LYTAC, since endocytosis does not require crossing the membrane barrier (which is complex due to the high molecular weight of the antibody) [25]. Conversely, CMA-based degraders are mostly chimeric peptides considering the recognition motif for HSPA8 is a peptidic motif. Thus, the key attention is directed toward the cellular delivery mechanism that will determine the chemical nature and the targeting characteristics of the drug.

### Small molecules

The discovery of small protein inhibitor molecules has been at the core of academic and private research for decades. The discovery of hits (whether by high-throughput screening or other methods) is followed by optimization steps that are greatly aided by structural studies (co-crystallization) or modeling of interactions, at the atomic level, between a target and its small molecule ligand. The TPD approach using small molecule-based chimeras is a promising first choice and strategy for selectively reducing drug concentrations [116]. Small molecule ligands have advantage of low molecular weight in addition to high stability, and fit within the classic drug design [33] (small molecule alone compared to heterobifunctional degrader that are in the “beyond rule of 5” space [117]) making easier to determine pharmacokinetics in the first step of drug development. Despite the use of small molecules for the POI ligand, the resulting conjugates generally extend beyond rule of five. Synthetic ligand can also be a classical non-covalent inhibitor/ligand or exhibit warhead used to covalently bind to the target protein and greatly enhance the complex degradation [61]. Despite these advancements, the field still faces significant challenges, particularly when it comes to targeting “undruggable” proteins – those that have proven difficult to inhibit using conventional small molecule approaches [118]. Traditional synthesis methods for small molecules can be limited in their ability to effectively engage these challenging targets and continue to be tedious in terms of synthesis and purification.

### Peptides

In response to these limitations, there has been a notable shift toward exploring alternative chemical entities, such as specific binding peptides. Peptides, which are short chains of amino acids, can offer unique advantages in targeting proteins that are otherwise considered undruggable. Their inherent ability to fold into specific three-dimensional structures with a conformational flexibility allows them to interact with protein surfaces in ways that small molecules may not be able to achieve [119]. Additionally, peptides can be engineered to enhance their stability, specificity, and affinity for their targets, making them promising candidates for therapeutic development using notably CPP (cell-penetrating peptide) [120]. These peptides offer the advantage of easy design with computational modeling and crystal structure of the protein of interest, along with an ease of chemical synthesis at low cost [121]. This exploration of peptide-based agents represents a significant evolution in drug discovery strategies, as researchers seek to expand the repertoire of available tools for targeting complex biological systems [122,123]. By integrating advanced molecular visualization techniques with innovative approaches to peptide design, the field is ready to make substantial progress in addressing some of the most challenging targets in modern medicine. The challenge will remain the inefficient cell permeability as peptides typically require delivery via nanoparticle, the short plasma half-life of peptides [124] and the cost of manufacture at a large scale compared to small molecules [125].

### Antibodies

Antibodies are specialized proteins produced by the immune system that exhibit a highly targeted affinity for specific proteins of interest [126]. This specificity allows them to bind effectively to their intended targets. However, antibodies possess certain limitations that can hinder their effectiveness in certain applications such as their relatively large molecular size. This size can impede their ability to permeate tissues effectively, especially when compared to smaller molecules, such as small-molecule drugs or peptides. The larger size of antibodies can restrict their diffusion through biological barriers, which may limit their therapeutic efficacy in certain contexts, particularly in solid tumors or other dense tissues [127]. In addition to issues related to size [52], antibodies are often characterized by low stability in various environments [58]. This instability can pose challenges in both storage and administration, necessitating careful handling and formulation strategies to maintain their integrity [128]. Others concern associated with the use of antibodies are the potential for immunogenic responses and their high cost of production and purification. While antibodies offer a powerful means of targeting specific proteins of interest due to their high specificity, their large molecular size, and elevated production costs present challenges that must be addressed [116]. Finally, a new generation of antibodies have been engineered for precise spatiotemporal control using programmable pH-dependent antibodies [65].

### Aptamers

Considering the shortcomings of the chemical groups featured above, aptamers have been proposed as a promising alternative [129]. These nucleic acid-based molecules, which can be engineered to bind specifically to a wide range of targets, including proteins, offer several distinct advantages [71]. One of the most significant benefits of aptamers is their low toxicity. Unlike many conventional drugs that can have off-target effects, aptamers are designed for high affinity and bind selectively to their designated target. This selectivity minimizes off-target effects, thereby reducing the risk of toxicity and making aptamers safer for therapeutic use. In addition to their low toxicity, aptamers exhibit reduced immunogenicity [129]. Because they are composed of nucleic acids, they are less likely to provoke an immune response compared to protein-based therapeutics or other biologics. Another key advantage of aptamers is their enhanced versatility for chemical modifications [70]. Aptamers exhibit a compact architecture that enhances their stability making them temperature and protease resistant [130]. Additionally, their compact form supports the creation of diverse delivery systems, such as nanoparticles, which can optimize the pharmacokinetics and delivery of the aptamer. Aptamers represent a versatile class of biomolecules that address many of the limitations associated with traditional chemical groups in drug development. Still, this chemical structure face issues related to low efficiency in cellular uptake due to their susceptibility to hydrolysis of nuclease and rapid clearance [131]. The binding affinity of aptamers can also be influenced by external factors, including pH, temperature, and salt concentration, making their performance dependent on the surrounding conditions [118].

## Design strategy

### Heterobifunctional degraders

**Figure 2.**
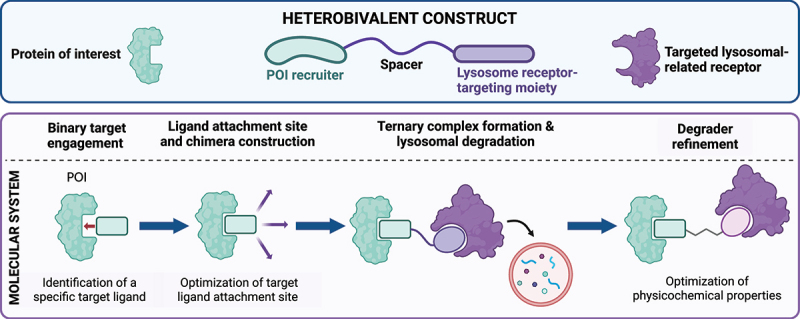
Structure and development stages of a heterobivalent degrader. The construction of a heterobivalent degrader takes into account the POI ligand, the spacer, and the lysosome receptor-targeting moiety. The development of the heterobifunctional construct typically goes through 4 phases and relies on the management of a range of properties.

Heterobifunctional degraders, including lysosomal-targeting technologies, represent an advanced strategy for targeted protein degradation. These bifunctional molecules consist of two distinct binding domains, or “warheads,” connected by a chemical linker (Figure 2). One domain binds to the protein/complex of interest, while the other recruits a component of the degradation machinery, such as an autophagy receptor or an LTR. This dual-binding mechanism drives target protein to the corresponding degradation pathway, ensuring its efficient breakdown. Unlike traditional small-molecule inhibitors, heterobifunctional degraders offer a broad range of applications and extensibility [132,133]. Similar to PROTACs, classic lysosomal degraders can employ heterobifunctional designs but recruit lysosomal receptors or autophagy components instead of E3 ligases [132,134]. The development and design of those lysosomal-based degraders follow a multi-step process, aimed at creating molecules capable of selectively directing target proteins to the lysosome for degradation (Figure 2). This targeted protein degradation process can generally be divided into four key stages, beginning with the identification of a ligand/binding moiety that interacts with the protein/complex of interest and the validation of a hit molecule [135]. These binding moieties can be derived from existing small molecules, antibodies, aptamers with a preference for those that exhibit high binding affinity, or novel discovery efforts.

The next critical step involves determining the optimal attachment site of the linker, which connects the target-binding moiety to the lysosomal-targeting domain. The linker vector design may be developed based on modeling and POI crystal structures with bound target ligand [121]. Pharmacophore modeling and docking can predict and showcase the accessibility of the binding pocket and attachment points preserving target protein binding. Structure-activity relationships may also be a technique, although this approach often relies on iterative optimization. Early designs prioritize maintaining high affinity and selectivity for the complex of interest while enabling trafficking to the lysosome [135].

A key component of lysosomal degraders is the lysosome-targeting binding element, which recruits the degradation machinery. Lots of lysosomal-based technologies rely on receptors such as IGF2R, TFRC, LC3 or HSPA8 among others (Tables 2 and 3), depending on the nature of the target complex and its location. Extracellular and membrane proteins are typically directed through the endocytosis-lysosomal degradation pathway, while cytosolic proteins and organelles will be preferably degraded via autophagy machinery. The choice of receptor also depends on factors such as cell-type specificity and target accessibility. For example, receptors highly expressed in certain tissues or cellular compartments can be strategically exploited to enhance degrader efficacy while minimizing off-target effects. Besides, the ternary complex formation and its management by degradation machinery needs to be assessed [26].

Once the target-binding ligand and lysosome-targeting component are identified, optimization becomes the focus for enhancing affinity and pharmacokinetic properties. Due to their large size and increased complex structure, lysosomal degraders often fall outside traditional drug-like parameters, such as Lipinski’s Rule of Five, leading to challenges like poor water solubility, high first-pass metabolism, and low oral bioavailability [9,117]. Addressing these limits requires strategic adjustments to the degrader’s molecular design with particular attention to the spacer [8]. The length of the spacer, in addition to the type of linker, plays a crucial role in influencing the degradation activity of theses degraders [136]. Maple’s research team developed an extensive database comprising over 400 published PROTACs to identify principles relevant to PROTAC design and the types of linkers employed. Flexible linkers, such as polyethylene glycol (PEG), alkyl and glycol chains are commonly used in early-stage development to demonstrate proof of concept [1] and are later refined [137]. Recently, Bemis et al. introduced a model for studying structure-activity relationships in PROTACS development concerning linear linker lengths, indicating that degraders featuring longer linkers are more likely to be effective in the initial design phase. After identifying a successful PROTAC, the linker length will be significantly reduced to determine the optimal length [1]. Trade-offs need to be considered between a longer linker offering better accessibility but risk of flexibility, disorganization, a higher molecular weight, and a smaller linker that may be excessively short. In this case, the two ligands may struggle to bind to their respective targets simultaneously due to steric hindrance, thereby preventing the formation of the ternary complex [136]. Nevertheless, linker length may be less critical in the design of lysosomal-degraders since no interactions are required between the protein of interest and the lysosome-targeting protein, as is the case for PROTAC. Concerning the absorption, distribution, metabolism, and excretion (ADME) parameters, it is crucial to evaluate the properties like metabolic stability, membrane permeability, oral bioavailability and systemic clearance, as early as possible in the optimization stage of the degrader [117,128,138]. Lysosomal degraders exceed traditional drug-like parameters described by Rule of Five, thereby design efforts continually focus on improving linker properties and on a more active passage through the membrane, and so enhancing compatibility with lysosomal pathways [139] using cell penetrating peptide CPP (amino acids with positively charged side chains like arginine) or cell membrane penetrating domain CMPD (TAT sequence) used as an inherent part of the linker [27,28,108,115].

Following the identification of lead degrader designs through discovery workflows, the subsequent phases generally involve conducting in vivo pharmacokinetic/PK and efficacy assessments utilizing appropriate preclinical models. These studies are essential for evaluating the biological activity of the identified compounds within a living organism [135,140]. The versatility of heterobivalent chimeras allows for the degradation of varied range of targets, from proteins, lipid droplets, protein aggregates to damaged organelles. The success of this approach hinges on the stability and compatibility of the ternary complex formed between the degrader, the target, and the recruited degradation component [26]. The major drawback to heterobivalent degraders is to frequently demonstrate the phenomenon known as the “hook effect.” This effect is characterized by a diminished degradation efficiency, which arises from an increased formation of binary complexes and an attendant decrease in the ratio of ternary complexes when the concentration of the degrader is either too low or elevated [141,142].

### Beyond classic chimera model

**Figure 3.**
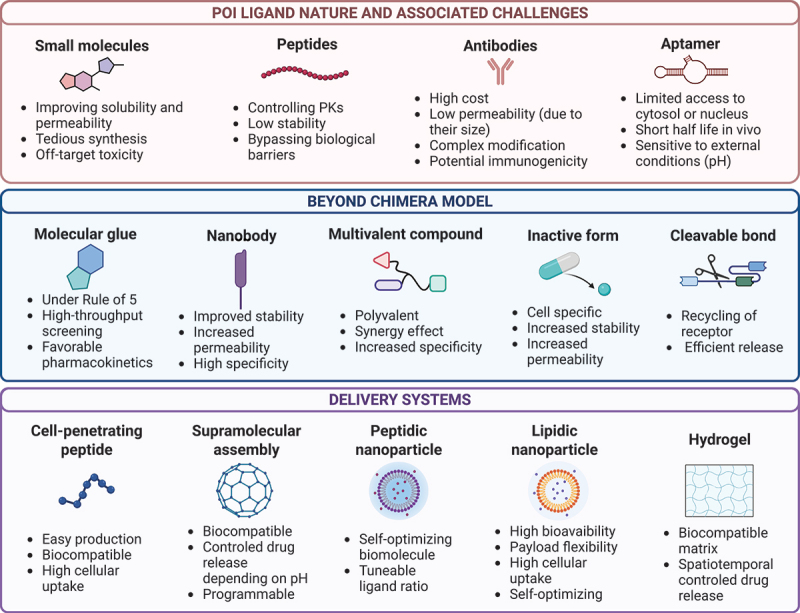
Nature of lysosome-targeting chimera scaffold and associated challenges; new development outside of heterobivalent construct and latest advancements in term of delivery systems developed for lysosomal-based degradation.

This limitation in the heterobivalent scaffold have prompted research teams to look beyond the chimera model (Figure 3) and aim to develop new generation of lysosomal-based degradation in term of delivery systems.

#### Molecular glues

Molecular glues, such as compounds targeting mutant HTT protein [33], offer a novel approach to targeted protein degradation by inducing interactions between a protein of interest and LC3 in autophagy-based degradation pathways. Unlike bifunctional degraders, molecular glues are single entities that stabilize protein-protein interactions by sitting at their interface [143], facilitating the recruitment of the target protein into the degradation pathway. Their small size allows them to pass the blood-brain barrier/BBB, making them especially attractive for treating central nervous system diseases and likely results in favorable pharmacokinetic properties such as oral bioavailability [144]. Additionally, molecular glues are compatible with high-throughput screening techniques, including small-molecule microarray-based methods [33], enabling the efficient identification of compounds that interact with both LC3 and the protein of interest. This screening capability significantly accelerates the discovery and optimization process. However, molecular glues are not without limitations. Their effectiveness relies on specific protein-protein interactions, which can make their identification highly challenging. In addition, their narrower range of applications compared to bifunctional degraders may limit their use to select targets. Their design is instrincally connected to the two proteins they glue together, thereby limiting the generalization of the molecular scaffold from one target to another. The primary challenge lies in the absence of a systematic, rational approach for identifying new molecular glues [4]. Despite these challenges, molecular glues remain a promising strategy for addressing diseases like Huntington disease, where protein accumulation plays a critical role, and their unique advantages position them as a valuable tool in drug discovery [33]. A growing number of molecular glue degraders have now been developed, most of which act by harnessing the ubiquitin-proteasome system [145,146]. Examples include CC-885, which promotes E3 ubiquitin ligase (CRBN)-mediated degradation of GSPT1 (G1 To S phase transition 1) [147], and indisulam, which triggers proteasomal degradation of RBM39 (RNA binding motif protein 39) [148].

#### Nanobody-based lysosomal degraders

Nanobody-based lysosomal degraders leverage the unique properties of nanobodies to target proteins for lysosomal degradation. Derived from camelid heavy-chain antibodies, nanobodies are significantly smaller than conventional antibodies, giving them exceptional stability, high tissue permeability, and the ability to bind otherwise inaccessible receptors [49]. The corresponding degraders often incorporate lysosomal-sorting sequences LSS [27,28], ligands [76] and even directly cell-surface lysosome-targeting receptors [100] to enable internalization and degradation of target proteins. Nanobody-based degraders can also achieve irreversible covalent binding to their protein of interest, enhancing degradation efficacy. Additionally, the “knobs-into-holes” method [78] for recombinant antibody production allows bispecific nanobody degraders to avoid common issues like light chain-heavy chain mispairing, ensuring more reliable engineering [76]. Moreover, both the SignalTAC and autophagy-targeting nanobody chimeras (ATNC) technologies exhibit a cell-penetrating peptide [100]: one a CPP [27] and one cyclic cell-penetrating deca-arginine (cR10*) [100], to help an efficient entry in the cell. While less immunogenic than full-length antibodies, potential immune responses must still be addressed with nanobody degraders. Moreover, they still display a short half-life [149] and a time-consuming process of generation [141]. In spite of these limitations and with their superior stability, specificity, and versatility [76] nanobody degraders represent an exciting approach for targeting extracellular and membrane proteins.

#### Multitarget compound

Trivalent degraders have come to light over the last few years, offering the capability to target and degrade multiple target proteins simultaneously through lysosomal pathways [57]. Unlike traditional bifunctional degraders, trivalent systems are designed with three distinct binding domains, presenting the ability to tether more than one protein of interest to the degradation machinery. This multivalent design allows for the simultaneous degradation of two or more targets, providing flexibility in modulating the degradation ratio between complex of interest. Such flexibility makes trivalent compounds a promising strategy for addressing complex diseases, as dual targeting may provide synergistic effects and could potentially enhance therapeutic efficacy while reducing the risk of drug-drug interactions [150]. One of the key advantages of trivalent degraders is their ability to improve targeting efficiency by binding multiple protein of interests (POIs) with high affinity [151,152]. This is achieved through the simultaneous engagement of multivalent aptamers or polyvalent antibodies [27,57,69], which has been widely exploited in other therapeutic contexts to enhance binding strength and degradation efficacy. In LYTAC constructs, the integration of trivalent targeting domains has shown promise in enhancing lysosomal trafficking and improving the overall therapeutic effect by triggering more efficient protein internalization and degradation [69]. The use of trivalent strategies can also help reduce off-target effects, as the increased specificity ensures that only cells expressing the desired combination of targets are affected. Tetravalent SignalTACs illustrate the potency of this approach [27]. By fusing peptide motifs LSS to both the heavy and light chains of antibodies, monoclonal antibody/mAb tetravalent SignalTACs exhibit significantly enhanced degradation activity compared to divalent versions, which modify only a single chain. Furthermore, recent construction proposed to modulate ligand ratios to enhance degradation efficiency. In the AptLYTAC technology developed by Duan et al., the ratio between the two targeting ligands can be varied in the multivalent AptLYTACs achieving higher efficiency than the monovalent counterpart [57]. The EndoTAC technology by Wang et al. utilizes peptide-decorated nanoparticles for lysosomal degradation, showing that increasing the ratio of targeting ligand allows polyvalent binding and so enhances cellular uptake and degradation efficiency [153]. Both approaches demonstrate how adjusting ligand ratios can significantly improve targeting specificity and lysosomal degradation. This increased activity highlights the critical role of multivalency in enhancing the degradation efficiency of lysosomal degraders. Moreover, the modular nature of trivalent compounds allows researchers to fine-tune the composition of the degrader, optimizing the balance between therapeutic potency and safety.

#### Inactive form

The concept of degrader with an inactive form, developed in particular by Fang et al., responds to the challenge of off-target effects by ensuring cell-selective membrane protein degradation. It is achieved thanks to the incorporation of a trivalent targeting mechanism designed to enhance specificity and minimize systemic toxicity [73]. In this approach, Logic-identification system endowed LYTAC (LogicTACs) molecules [73] are rendered inactive during circulation by using a duplex DNA structure that “locks” the ligand recognition sites for both the protein of interest and lysosome-targeting receptor by self-assembling double strand DNA. Upon encountering cancer-specific markers, here the epithelial cell adhesion molecule (EPCAM/CD326), the LogicTAC undergoes activation. This process exposes the binding sites, allowing the LYTAC to tether the protein of interest. In a second time, LogicTAC binds to the lysosome targeting receptor, this way, this dual-input will ensure that the endosomal-lysosomal-degradation machinery is only engaged in the presence of specific markers. So, this nucleic acid-based LYTAC remains inactive in circulation, significantly improving the precision of the protein of interest degradation while avoiding nonspecific internalization. Another technology called nano-ATTEC [95] by Xu et al. used a polymeric micelle with an hydrophilic and neutral form at physiological pH and a positively charged form (notably poly[β-aminoester] polymer) in the acidic tumor microenvironment (TME) making cellular uptake efficient. Nano-ATTEC facilitates self-adaptive targeting of the acidic TME, improving the cell selectivity and the delivery of the degrader in the cytosol.

#### Cleavable bonds

Zhang et al. reported a cleavable linker strategy into the design of transferrin receptor-targeting chimeras known as TransTAC to enhance membrane protein degradation by controlled release of the target protein in the endosomes [77]. By incorporating a CTSB (cathepsin B)-sensitive linker between the target-binders and TFRC binders, the construct allows cleavage within early endosomes by effectively disconnecting the target protein from TFRC. NanoCLY technology developed by Lin et al., proposed another cleavable linker strategy based on a disulfide linker sensitive to glutathione (GSH). GSH concentrations is higher in the TME, resulting in a TME-cleavable LYTAC linker [60]. These modular designs overcome limitations seen in earlier approaches where persistent receptor binding hindered lysosomal processing and features the utility of cleavable linkers in improving degrader efficacy and intracellular routing.

### Delivery advancement

While technologies such as lysosome-targeting chimeras have demonstrated significant therapeutic potential, efficient delivery mechanisms remain critical to their success. The efficacy of large lead compounds is frequently constrained by their low permeability across cell membranes. To address this limitation, researchers are not only exploring structural modifications of therapeutic protein drugs but are also concentrating on the creation of novel formulations and delivery systems [135]. These efforts are designed to minimize nonspecific interactions with biological entities while facilitating a controlled release of the drug at specific disease locations, both in terms of spatial and temporal dynamics [9]. The recent introduction of different delivery systems such as cell-penetrating peptides, supramolecular assembly, nanoparticles, and encapsulation techniques via hydrogel presents in Figure 3 offers a multiplicity of assets and will likely improve POIs targeting along with efficient therapeutic strategies.

#### Cell penetrating peptides

Cell-penetrating peptides facilitate the intracellular delivery of therapeutic compounds trough cell membranes and are now commonly used in the development of degraders [27–29,34,98,99,108,114,115]. Advanced CPP designs, such as supercharged polypeptides/SCPs like K20 [154], exhibit high cellular uptake efficiency and endosomal escape capabilities. These peptides can deliver a variety of cargoes, including proteins and nucleic acids, directly into the cytosol, enhancing the therapeutic potential of lysosomal degraders [120]. The cyclic cell-penetrating deca-arginine (cR10*) proposed by He et al. with the autophagy-targeting nanobody chimeras (ATNC), offers a unique advantage as it uses a cell-permeable phagobody with a cleavable link via reduction reaction [100]. After crossing the cell membrane, the disulfide bridge can effectively be cleaved and release the cR10* part within the intracellular environment. The integration of CPPs into delivery systems, such as extracellular vesicles [155] and nanoparticles [154], further amplifies their efficacy.

#### Supramolecular assembly

Supramolecular assembly uses dynamic, non-covalent interactions to create elaborate delivery systems in the form of nanoscale particles. These assemblies are highly programmable and demonstrate promising bioavailability and biocompatibility, along with simple preparation. These features make them promising candidates for complex therapeutic applications. For instance Wang et al, self-assembled Nano-AUTAC incorporate lysosome-targeting motifs methotrexate/MTX in combination with a guanine derivative and competitive Indoleamine 2,3-dioxygenase/IDO inhibitor chimera via hydrogen bonding [84]. The platform used guanine derivative capability to recognize other nucleotide-like drugs to form supramolecular nanoparticles and so helped counteract the high polarity of AUTAC molecules. Nano-AUTAC demonstrated complete drug loading efficiency and pH-responsive release, triggered specifically by the acidic lysosomal environment [84]. Supra-LYTAC by Kim et al. featured a supramolecular self-assembly of two functional peptides. This self-assembly form nanofibers thanks to π-π stacking hydrophobic interactions between Pyrene-Phe-Phe-based peptides [63]. These two technologies not only improved cellular uptake but also enhanced lysosomal delivery and in the case of Nano-AUTAC [84] potential synergistic therapeutic efficacy, illustrating how supramolecular engineering can optimize both degrader targeting and pharmacodynamics.

#### Nanoparticles

Spherical Nanoparticles provide a versatile platform for the delivery of lysosomal degraders [156]. Multiple types of nanoparticles exist, depending on the nature of the particle, including polymeric nanoparticle [60,95,111,153], lipid-based nanoparticle [112,157], supramolecular assembled-particle [84] and even inorganic nanoparticle. Their tunable size, self-assembly ability [111], and capacity to transport efficiently diverse molecules into cells with low cytotoxicity [158] make them ideal for delivery purposes. The transport can be by encapsulation of the degrader [112] or by adding a hydrophobic motif, resulting in a self-assembling nanoparticle [60,111,153]. Wang et al, with split-and-mix chaperone-mediated autophagy-based degrader SM-CMAD [111], by bypassing linker optimization steps, have a simpler developing process compared with traditional heterobifunctional degraders. Additionally, these particles demonstrated the ability to adjust the ligand ratios to refine the POI degradation [111,153,157]. More than helping the delivery to the cell vicinity, the mixed-shell polymeric micelle (MSPM) by Xu et al. [95] possess a pH-responsive polymer, neutral at a physiological pH and positively charged in the acidic TME that can greatly enhance the delivery in the cell cytosol. Another degrader named CMATAC, allows the encapsulation of the compound in lipidic nanoparticles composed of an ionizable cationic lipid using a micromixer chip [112]. In the continuity of spherical nanoparticles, also exist extracellular vesicles. They are emerging as natural delivery vehicles due to their inherent biocompatibility and ability to cross biological barriers. Recent advancements by Noguchi et al, include modifying extracellular vesicles (specifically exosomes) membranes with cell-penetrating peptides derived from CAMP/CAP18 [155], which enhance cellular uptake via macro-pinocytosis. These engineered extracellular vesicles demonstrate improved targeting and cytosolic delivery capabilities, positioning them as a promising vehicle for the delivery of lysosomal degraders.

#### Hydrogel

Finally, encapsulation techniques including hydrogel provide a controlled and protective environment for lysosomal degraders, enhancing their stability and delivery efficiency. The use of nucleic acid hydrogels, such as the LYTAC Plus platform [69], exemplifies this approach. This hydrogel integrates lysosome-targeting and gene silencing compounds by encapsulating therapeutic agents within a biocompatible matrix. The hydrogel degrades into smaller particles upon enzymatic exposure, facilitating the controlled release of the lysosomal-based degrader.

## Conclusions and perspectives

Lysosomal-based degraders have entered a phase of rapid conceptual expansion, with their mechanisms now extending across distinct branches of the lysosomal pathway. Their appeal lies in a combination of features that set them apart from proteasome-directed approaches. These degraders have, with their promising versatility, potential applications across a range of diseases, including cancer, neurodegenerative disorders, and genetic conditions. They can be tailored for tissue specificity through the use of receptors with restricted patterns of expression, thereby offering a degree of selectivity that reduces systemic toxicity. In addition, lysosomal-based degraders are not confined to a single class of proteins and extend the range of targets accessible through PROTACs. They can direct extracellular factors, membrane receptors, as well as cytosolic substrates, toward lysosomal clearance. Furthermore, these degraders extend beyond protein targeting, encompassing nucleic acids, dysfunctional organelles, lipid droplets, and pathogens. Parallel to these biological advances, considerable progress has been made in delivery technologies, with novel carriers and conjugation strategies transforming these degraders into increasingly sophisticated systems. The possibility of designing multifunctional platforms by engaging multiple receptors/proteins that integrate degradation with targeted delivery and therapeutic modulation deepens their potential as versatile tools.

LYTACs represent a rapidly evolving method for targeted protein degradation (TPD) and hold significant therapeutic promise [159]; however, they face limitations concerning optimization, safety, and, crucially, the recycling of materials [160]. Quantification and evaluation of the enzyme kinetics and potencies of lysosome-mediated degraders present greater difficulties than those for PROTACs. This increased complexity arises from the former’s interaction with a broader array of cellular components, including proteins and lipids, in contrast to the more straightforward engagement of PROTACs with well-characterized E3 enzymes and proteasome [161]. Lysosomes are present in nearly all animal cells, as a result, the process of lysosomal protein degradation is theoretically applicable across a wide range of cell types [25]. While numerous PROTAC-based molecules have successfully entered clinical development, lysosomal-targeting degraders remain largely confined to the preclinical stage [160]. This discrepancy reflects several unresolved challenges unique to lysosomal-based strategies. First, many lysosomal degraders such as LYTACs, AbTACs, and nanobody-based platforms exhibit high molecular weights and structural complexity, which compromise pharmacokinetic profiles and hinder cellular penetration [127]. In addition, technologies involving antibodies, recombinant fusion proteins, or aptamer-based constructs carry inherent risks of immunogenicity. These concerns are amplified in repeated dosing regimens, which are often required for sustained protein knockdown. Moreover, lysosomal degraders typically rely on specific receptors such as IGF2R, IL2, or TFRC that vary in expression across tissues and individuals, raising concerns about target accessibility and therapeutic consistency. A further limitation is the complexity of the degradation mechanism itself. Unlike PROTACs, which involve intracellular ubiquitination and proteasomal processing, lysosomal pathways require multiple sequential steps including internalization, endosomal trafficking, lysosomal fusion, and enzymatic degradation for the endocytosis-lysosomal-based degraders. This multistep process complicates pharmacodynamic modeling and in vivo monitoring, making it harder to evaluate efficacy and optimize dosing strategies in animal models and clinical settings.

Lysosome-based degraders represent a rapidly evolving method for targeted protein degradation (TPD) and hold significant therapeutic promise. To fully harness the potential of lysosome-mediated degraders, several forward-looking strategies have been developed, each designed to address current challenges while opening new avenues of application. This new generation of degraders shows improvements in delivery systems, tackling permeability challenges, ensuring effective delivery of large therapeutic agents [109]. Innovations in formulation, added complexity to the scaffold with cleavable linker or prodrug, and delivery technologies have enhanced cellular uptake and potent transport up to the target.

## Abbreviations

AbTACs: antibodies-based PROTAC ;
AptLYTACs: aptamer-based LYTACs;
ATNC: autophagy-targeting nanobody chimeras;
ATTECs: autophagy-tethering compounds;
AUTACs: autophagy-targeting chimeras (S-guanylation-based autophagy inducer);
AUTOTACs: autophagy-targeting chimeras (SQSTM1/p62-activating autophagy inducer);
BIACs: bispecific aptamer chimeras;
CMA: chaperone-mediated autophagy;
CMATAC: chaperone-mediated autophagy targeting chimeras;
CMPD: cell membrane penetrating domain;
CPP: cell-penetrating peptide;
cR10*: cyclic cell-penetrating deca-arginine;
CTM: chaperone-targeting motif;
GalNAc: N-acetylgalactosamine;
GlueTACs: glue-targeted chimeras;
KineTACs: cytokine receptor-targeting chimeras;
LDs: lipid droplets;
LMPs: lysosomal membrane proteins;
LogicTAC: logic-identification system endowed LYTAC;
LSS: lysosome sorting sequence;
LTRs: lysosome targeting receptors;
LYMTACs: lysosome membrane targeting chimeras;
LYTACs: lysosome targeting chimeras;
M6P: mannose-6-phosphate;
MA: macroautophagy;
Mi: microautophagy;
PBD: protein binding domain;
PEG: polyethylene glycol
POI: protein of interest
PROTAC: proteolysis-targeting chimeras;
PSMLTACs: peptide-mediated small molecule lysosome-targeting chimeras;
SignalTAC: signal-mediated lysosome-targeting chimera;
TME: tumor microenvironment;
TPD: targeted protein degradation;
TransTACs: transferrin receptor-targeting chimeras;
UPS: ubiquitin-proteasome system.
